# Ruminally Protected Isoleucine, Leucine, Methionine, and Threonine Supplementation of Low-Protein Diets Improved the Performance and Nitrogen Efficiency of Dairy Cows

**DOI:** 10.3390/ani15091210

**Published:** 2025-04-24

**Authors:** Xiaoli Qin, Xueyan Lin, Mark D. Hanigan, Kai Zhao, Zhiyong Hu, Yun Wang, Qiuling Hou, Zhonghua Wang

**Affiliations:** 1 Ruminant Nutrition and Physiology Laboratory, College of Animal Science and Technology, Shandong Agricultural University, Tai’an 271018, China; qinxiaolicau@outlook.com (X.Q.);; 2 Department of Dairy Science, Virginia Tech, Blacksburg, VA 24061, USA; 3 Faculty of Engineering and Applied Science, University of Regina, Regina, SK S4S 0A2, Canada

**Keywords:** rumen-protected amino acid, nitrogen balance, milk performance, low protein diets, mTORC1

## Abstract

This study evaluated the effects of supplementing rumen-protected methionine, threonine, isoleucine, and leucine to low-protein diets on lactating dairy cow performance. Sixty Holstein cows were assigned to one of four dietary treatments in a 9-week randomized complete block design: positive control (16% crude protein diet; 16% CP), negative control (12% CP), 12% CP plus the four essential amino acids (12% CP + EAA), and 14% CP supplemented with the four EAA (14% CP + EAA). The milk protein yield was significantly decreased in the 12% CP group compared to the 16% CP group but was restored to comparable levels with EAA supplementation of both the 12% and 14% CP diets. Dietary nitrogen intake and urinary nitrogen excretion both increased with higher dietary CP levels. Nitrogen utilization efficiency in milk was significantly improved by EAA supplementation, with the highest efficiency observed in the 12% CP + EAA treatment (39.0% vs. 33.3% in the 16% CP diet). Plasma urea levels increased with elevated dietary CP and EAA supplementation. Moreover, EAA supplementation significantly elevated venous methionine levels and showed a tendency to increase venous leucine levels. Additionally, compared to the negative control, EAA supplementation increased concentrations of glucagon and prolactin (*p* < 0.05). EAA supplementation of low-protein diets, particularly the 14% CP diet, improved the dietary protein efficiency of lactating cows without a concomitant decrease in milk protein yield.

## Simple Summary

Driven by potential economic and environmental benefits, researchers and farmers are strongly interested in developing technologies to improve dietary nitrogen efficiency in animal production. However, in modern dairy production, the efficiency of conversion of dietary nitrogen to milk protein is approximately 25%, which is much lower than the 50% efficiency of nitrogen conversion in modern broiler production, and there is potential for further improvement. This research aimed to explore the impact of low-protein diets supplemented with specific amino acids on the nitrogen utilization efficiency and milk production of dairy cows. The findings showed that adding specific amino acids to low-protein diets enabled cows to produce milk yields and milk protein levels comparable to those observed with high-protein diets. Simultaneously, cows demonstrated improved nitrogen efficiency and decreased nitrogen excretion through manure and urine. This study suggests that strategically reducing dietary protein levels while supplementing with specific amino acids may serve as an effective strategy to enhance nitrogen efficiency and maintain milk production. This approach is anticipated to promote more sustainable and environmentally friendly dairy farming.

## 1. Introduction

Researchers and farmers maintain a strong interest in developing techniques and practices to enhance dietary nitrogen efficiency in animal production, driven by their potential economic and environmental benefits. In modern dairy cow production, the dietary nitrogen conversion efficiency into milk protein is approximately 25% [1], which is lower than the roughly 50% observed in modern broiler production [2]. This discrepancy highlights the differences in nitrogen utilization between ruminants and monogastric animals. In dairy cows, a substantial portion of dietary nitrogen is lost as urinary and fecal nitrogen rather than being utilized for milk protein synthesis. This inefficiency is partly due to the complex digestive system of ruminants and the extraction and metabolism of absorbed amino acids within the mammary gland. By contrast, broiler chickens have a more efficient nitrogen conversion due to their simpler digestive systems and the ability to utilize dietary amino acids more effectively for growth and protein synthesis. Therefore, there is considerable potential to improve nitrogen conversion efficiency in dairy cows through targeted nutritional strategies.

Recent research has demonstrated that the nitrogen utilization efficiency of lactating cows can be improved by feeding low-protein diets [3]. Reducing dietary crude protein (CP) can also decrease nitrogen emissions from manure and urine, promoting more sustainable dairy production practices. However, this approach typically reduces milk or milk protein yields, rendering it economically unviable for dairy farmers. To address this, a prior study explored enhancing the dietary nitrogen efficiency of lactating Holstein cows by supplementing a low-protein diet with lysine (Lys), methionine (Met), and leucine (Leu), activators of the mammalian target of rapamycin complex 1 (mTORC1), applied individually or in combination [4]. Essential amino acid (EAA) supplementation treatments increased the milk true protein yield to a level comparable to that of the positive control. Additionally, an in vitro study indicated that the mTORC1 signal pathway in the mammary gland was activated by several EAAs rather than a single optimal EAA profile [5]. Therefore, combining a low-protein diet with mTORC1-stimulating EAAs emerges as a promising strategy for improving dietary nitrogen efficiency, warranting further investigation.

Milk protein synthesis is a complex biological process that is influenced by a variety of factors in the lactating cow. Internal physiological factors, such as energy supply and hormonal status (e.g., insulin, growth hormone), influence nutrient availability for the mammary cell. External factors, including farm environmental circumstances and management techniques (e.g., diet stability and milking frequency), also affect milk protein yield. Additionally, the cow’s breed and genetic characteristics play a role in the synthesis of milk protein. Among these factors, mTORC1 acts as a vital nutrient-sensing signal, which senses a variety of internal and external signals, encompassing cellular energy status, nutrient availability, growth regulators, and stress [6]. The amino acids that stimulate mTORC1 activity appear to be cell type-specific [7]. In the mammary tissue of lactating cows, it was identified that mTORC1 signaling was affected by Ile, Leu, Met, and Thr [4]. These results were further demonstrated in lactating rats, where individual supplementation of these four EAAs to a low-protein diet increased the phosphorylation levels of mTOR and eIF4E-binding protein 1 (4eBP1), with increased litter weight gain observed [8]. A subsequent study with mid-lactation cows confirmed that supplementation of ruminally protected Met, Thr, Leu, and Ile to a low-protein diet stimulated mammary mTORC1 signaling and recovered milk protein yield [9].

Although supportive evidence exists in the above literature, research has shown that mammary mTORC1 signaling responses to specific EAAs may be adaptive and temporary [10], potentially limiting the practical use of mTORC1-stimulating EAAs to enhance the dietary nitrogen efficiency of lactating cows. We hypothesized that supplementing low-protein diets with mTORC1-stimulating EAAs, specifically Met, Thr, Leu, and Ile, would enhance milk protein synthesis in lactating cows fed a low-protein diet. Based on this, a relatively large-scale feeding trial was carried out in the present study. The objectives of this study were to evaluate the effects of rumen-protected Met, Thr, Ile, and Leu supplementation of low-protein diets on production performance and nitrogen efficiency in Holstein cows and possible effects of the practice.

## 2. Materials and Methods

### 2.1. Animals and Experimental Design

The protocol was approved by the Shandong Agricultural University Laboratory Animal Welfare and Animal Experimental Ethical Committee (No. 2019-DG-0524). The experiment was conducted on a commercial farm.

Sixty multiparous lactating Holstein cows, averaging 629 ± 46 kg body weight and 119 ± 26 days in milk, were randomly assigned to four treatments (N = 15/treatment). The cows were placed in four separate pens, and the trial was conducted for 9 weeks. In sequence, 15 additional stalls equipped to conduct individual animal intakes were used to assess the dry matter intake (DMI) for each of the four groups. The offered diets and any refusals were tracked and documented for 7 successive days to calculate the DMI of each cow. The cows had free access to water and were milked three times daily at 0900, 1630, and 0030 h. Diets were prepared as TMR and delivered 3 times daily at 0800, 1530, and 2330 h with a daily amount allowing for about 10% refusal.

As presented in Table 1, three basal iso-energetic diets containing 12%, 14%, or 16% CP, respectively, were formulated according to the Nutrient Requirement of Dairy Cattle by the National Academies of Sciences, Engineering and Medicine (NASEM, 2021) [11]. An EAA mixture was prepared by mixing commercially purchased ruminally protected methionine (RP-Met), threonine (RP-The), leucine (RP-Leu), and isoleucine (RP-Ile) (King Tech. Feed Co. Ltd., No. 8, Ginkgo Rd., Hangzhou, China; Table 1). Cows in each treatment received one of the three basal diets with or without EAA supplementation. The four treatments were therefore 16% CP diet (positive control; 16% CP), 12% CP diet (negative control; 12% CP), 12% CP diet plus the four EAAs (12% CP + EAA), and 14% CP diet plus the four EAAs (14% CP + EAA), respectively. The daily supplementation with EAAs was equivalent to that in the amount of casein required to elevate the dietary CP of the 12% and 14% CP diets to that of the 16% diet for a cow ingesting 25 kg of DM per day (Table 1). The supplemental EAAs were top-dressed to the freshly dispatched TMR. The duodenal flow of each essential AA was estimated according to NASEM [11] (Table 2). Ruminal escape of the four RP-EAAs was assessed using an in situ experiment [9]. The intestinal digestibility of the four RP-EAAs was estimated using a three-step in vitro method [12].

### 2.2. Sampling and Analysis

Sampling was performed after a two-week adjustment period. Fresh TMR and daily refusals were collected over four consecutive days weekly, dried at 65 °C for 48 h, pooled by week, and stored at −20 °C for later analysis of dry matter (DM), CP, neutral detergent fiber (NDF), acid detergent fiber (ADF), ash, and acid insoluble ash (AIA).

Milk was sampled on two successive days each week. The samples gathered from the morning, afternoon, and night milking were pooled per cow and day. Potassium bichromate was used to preserve milk samples, which were then stored at 4 °C for subsequent analysis of milk fat, protein, lactose, total solids, and milk urea nitrogen (MUN).

Blood samples were drawn from the caudal vein on the first day of the 7th and 9th weeks, respectively, before the morning feeding. Blood, amounting to ten milliliters, was taken into a heparinized tube and subsequently analyzed for amino acids, nitric oxide, total protein, urea, insulin, glucagon, insulin-like growth factor (IGF-I), growth hormone (GH), and prolactin concentrations. Additional blood samples were drawn into NaF-containing tubes to analyze glucose. All samples were quickly placed on ice, centrifuged at 1500× *g* for 20 min, and then stored at −80 °C.

Urine samples were sampled by vulva stimulation and fecal grab samples were collected at 4 h intervals for a total of 6 samples, respectively, per cow in sampling days of the 7th and 9th week. Urine samples were mixed per week and cow for later analyses of CP, urea nitrogen, and creatinine concentrations. Fecal samples gathered from each cow and week were composited, dried at 65 °C, and then analyzed for DM, CP, NDF, ADF, ash, and AIA contents.

For analyses of TMR, refusals, and fecal samples, DM and CP content were determined according to the previous procedure [13]. The contents of NDF and ADF were measured using amylase and sodium sulfite [14]. Ash content was determined according to the method described by Yang and Beauchemin [15]. The apparent digestibility of the whole digestive tract was calculated using AIA in TMR and feces as an internal marker.

The creatinine and urea contents in urine samples were measured by using kits (JC Tech Co., Ltd., Nanjing, China). The calculation of daily urine output followed the previous procedure [16]:

*Urine*, (L/d) = BW (kg) × 29 mg creatinine/kg BW/d/*urinary creatine* (μmol/L)/113.1 (g/mol)

Urine output and concentrations of nitrogen and urea in urine were used to calculate the daily total urinary nitrogen and urea nitrogen (UUN) excretion.

Plasma amino acid concentrations were measured using an automated amino acid analyzer (U3000; Thermo Fisher, Shanghai, China). Plasma total protein, glucose, and urea were measured with an automated biochemical analyzer (7020; Hitachi, Tokyo, Japan) with commercially purchased kits (Sichuan Maker Bio Co., Ltd., Chengdu, China). Plasma nitric oxide and urea nitrogen were determined using the nitrate reductase method with commercially purchased kits (Jiancheng Bio Co. Ltd., Nanjing, China). All ELISA data were recorded by a Synergy hybrid spectrophotometer (BioTek, Winooski, VT, USA). The levels of plasma insulin, glucagon, IGF-1, GH, and prolactin were determined by an r-911 fully automatic immune counter (University of Technology Industrial Corporation of China, Nanchang, China) with commercially available kits (JD Biochemical Engineering Co. Ltd., Tianjin, China).

### 2.3. Statistical Analysis

Data were analyzed utilizing the PROC MIXED procedure of SAS (version 9.1; SAS Institute Inc., Cary, NC, USA). The statistical model employed was as follows:

Y_ijk_ = μ + τ_i_ + W_j_ + τW_ij_ + e_ijk_,

where Y_ijk_ was the dependent variable, μ was the overall mean, τ_j_ was the fixed effect of the jth treatment (16% CP, 12% CP, 12% CP + EAA, 14% CP + EAA), W_j_ was the fixed effect of the sampling week (7th week and 9th week), τW_ij_ was the random effect of the interaction of treatment and sampling week, and e_ijk_ was the random error term, which was assumed to be normally distributed. All data are presented as least squares means. Statistically significant differences among treatments were observed at *p* ≤ 0.05. Differences at 0.05 < *p* ≤ 0.10 were regarded as a trend toward significance.

## 3. Results

### 3.1. Predicted EAA Supply

Table 2 presents the predicted duodenal flows of digestible EAAs (NASEM, 2021) [11]. For the 16% CP diet, the duodenal flow of Met, Thr, Ile, and Leu represented 2.20, 5.23, 5.83, and 9.20% of metabolizable protein (MP), respectively. The estimates for duodenal flows of digestible Met, Thr, Ile, and Leu from the 12% CP diet were 2.26, 5.23, 5.71, and 9.54% of MP, respectively. EAA supplementation of the 12% CP diet elevated the duodenal flows of Met, Thr, Ile, and Leu to 2.61, 6.19, 6.91, and 10.9% of MP, respectively, and supplementation of the 14% CP diet elevated the duodenal flows to 2.38, 5.65, 6.31, and 9.95% of MP, respectively. The duodenal flows of digestible Met, Thr, Ile, and Leu for the 12% CP + EAA treatment were restored from 42, 97,106, and 177 g/d, respectively, to the level of 16% CP treatment (Met:51, Thr:121, Ile:135, and Leu:213 g/d).

### 3.2. Dietary Crude Protein Levels

The observed dietary CP concentrations of the three experimental diets (16% CP, 14% CP, and 12% CP) were 15.99%, 14.70%, and 12.40%, respectively (Table 1), due to efforts to maintain similar energy levels across the three diets. For cows receiving the 14% CP + EAA and 12% CP + EAA diets, 122.35 g/d and 242.34 g/d of EAAs were respectively supplemented to each cow, which elevated the calculated dietary CP concentrations to 14.7% and 12.4% (Table 3).

### 3.3. Nutrient Intake and Digestibility

There were no significant differences in DM and OM intakes across the treatments (Table 3). The CP intakes increased with dietary protein level and EAA supplementation (*p* < 0.01), except for the intakes of the 16% CP and 14% CP + EAA treatments, which did not differ (*p* = 0.19). Cows receiving the 12% CP + EAA treatment exhibited the highest NDF and ADF intakes, which were greater than those of cows receiving the 16% CP treatment (*p* < 0.05) and had a greater trend than those receiving the 14% CP + AA treatment (*p* < 0.1).

The apparent digestibility of CP throughout the whole tract was influenced by CP intake. For treatments with higher CP intakes, the apparent CP digestibility was also higher (*p* < 0.05). More grass hay was included in the roughage of the two 12% CP diets, which could be discerned from the lower NDF digestibility for cows receiving those treatments (*p* < 0.05). The ADF digestibility of the 12% CP + EAA treatment was also significantly decreased compared to that of the 16% CP treatment (*p* < 0.05).

### 3.4. Milk Yield and Composition

The milk yield was higher in cows fed the 16% CP positive control diet than in those fed the 12% CP negative control diet (Table 4). Supplementing the four EAA mixture to the 12% CP diet significantly increased the milk yield and tended to increase the production of energy-corrected milk (ECM) (*p* = 0.06). There were no significant differences in DMI among the 16% CP, 12% CP + EAA, and 14% CP + EAA treatments. Both milk protein, % and milk protein, kg/d were higher in the 16% CP treatment than in the 12% CP treatment (*p* < 0.01). Supplementing EAAs to the 12% CP diet elevated the milk protein yield (*p* < 0.05) but not milk protein % (*p* > 0.1). The milk protein % was lower in cows fed the 12% CP + EAA diet compared to those fed the 16% CP or 14% CP + EAA diets (*p* < 0.05), but the milk protein yield of the three treatments was similar (*p* > 0.05). Treatments did not affect milk fat % or yield (*p* > 0.05). The lactose yield was lower in cows fed the 12% CP diet than in those fed the other three diets (*p* = 0.05). The concentration of milk urea nitrogen (MUN) was elevated with dietary CP level and EAA supplementation (*p* < 0.01). Cows fed the 12% CP + EAA treatment exhibited the highest conversion efficiency of intake nitrogen into milk protein (*p* < 0.05; Table 5), whereas cows fed the 16% CP treatment had the lowest nitrogen utilization efficiency (*p* < 0.05).

### 3.5. Nitrogen Balance

Dietary nitrogen intake increased with dietary CP level and EAA supplementation (*p* < 0.01) (Table 5). Cows receiving the 12% CP diet secreted less milk nitrogen (*p* < 0.01) than cows receiving the other treatments, and the milk nitrogen secreted was similar across the other three treatments, except that the milk nitrogen (N) for the 12% CP + EAA treatment tended to be reduced compared to that of the 14% CP + EAA treatment (*p* = 0.10). Urinary nitrogen excretion increased with dietary CP level and EAA supplementation (*p* < 0.01), and only the difference between 12% CP and 12% CP + EAA treatments was statistically insignificant (*p* > 0.05). Fecal nitrogen excretion did not significantly vary between the treatments. Cows receiving the 16% CP diet had the lowest conversion efficiency of intake nitrogen into milk (*p* < 0.05), and the nitrogen utilization efficiency was significantly increased by EAA supplementation, especially in the 12% CP + EAA treatment. Cows receiving this treatment ingested less dietary protein (Table 3, *p* < 0.01), produced similar milk protein (Table 4, *p* = 0.19), and hence had higher dietary nitrogen efficiency (Table 5, *p* < 0.01) in comparison with those receiving the positive control treatment. The estimated nitrogen balance for cows with the 16% CP diet was slightly positive and was negative for cows with the other three treatments.

### 3.6. Plasma Free Amino Acids

For the four supplemented EAAs, plasma Thr was quite stable with no differences across treatments (Table 6). Cows receiving the 12% CP treatment had lower levels of plasma Ile, Leu, and Met than those receiving the 16% CP treatment (*p* ≤ 0.01). Supplementing the four EAAs to the 12% CP treatment significantly elevated plasma Met (*p* ≤ 0.01), which was restored to a level similar to that of the 16% CP treatment. Plasma Leu was numerically elevated (*p* = 0.16) and plasma Ile tended to be elevated (*p* < 0.1) by supplementation with the four EAAs. The plasma levels of Ile, Leu, and Met did not differ significantly between the 14% CP + EAA and 16% CP treatments, nor between the 14% CP + EAA and 12% CP + EAA treatments.

The plasma concentrations of the other five measured EAAs did not significantly differ between the 12% CP and 12% CP + EAA treatments and between the 14% CP + EAA and 16% CP treatments. Plasma Lys, Val, Phe, and His but not Arg were lower for the 12% CP treatment compared to the 16% CP treatment (*p* < 0.05). The plasma concentrations of all five of the other EAAs were lower (*p* < 0.05) or tended to be lower (His) (*p* < 0.1) for the 12% CP + EAA treatment than those of the 16% CP treatment. When the two EAA-supplemented treatments were compared, plasma His and Arg were lower (*p* < 0.05), and plasma Val tended to be lower for the 12% CP + EAA treatment than those of the 14% CP + EAA treatment (*p* < 0.1).

The plasma concentrations of the determined non-essential amino acids (NEAAs) were generally not affected by treatment except for Gly and Tyr. Plasma Gly was reduced (*p* < 0.05) for the 12% CP and 12% CP + EAA treatments and tended to decrease (*p* < 0.1) for the 14% CP + EAA treatment in comparison with the 16% treatment. The plasma Tyr concentration was lower for the 12% CP + EAA treatment (*p* < 0.05) compared to the positive control (16% CP).

### 3.7. Plasma Metabolites and Hormones

Plasma levels of total protein, glucose, nitric oxide, growth hormone, and IGF-I were not significantly altered by the treatments (Table 7). Plasma urea increased with dietary CP level and EAA supplementation as expected, which differed significantly between all of the compared treatment pairs (*p* < 0.01), except for that between 14% CP + EAA and 16% CP treatments (*p* > 0.05). An interesting finding was that feeding the 12% CP + EAA diet depressed plasma insulin in cows, which was decreased compared to that when feeding any of the other three diets (*p* < 0.05), and elevated plasma glucagon and prolactin, which were higher than that when feeding the 12% CP diet (*p* < 0.05) and tended to be higher than that when feeding the 16% CP diet (*p* < 0.1). The plasma levels of the three hormones did not show significant differences across the other three treatments. The reason that the 12% CP + EAA treatment is specific is an issue worthy of further investigation.

## 4. Discussion

Met, Ile, Leu, and Thr have been shown to stimulate mTORC1 signaling in mammary epithelium cells both in vitro [7] and in vivo [8]. The aim of the present study was therefore to evaluate whether these four EAAs could be used in practice to improve dietary protein efficiency in lactating cows. Milk protein yield is influenced by various factors, including genetic context and farm practice, but we focused on EAA supplementation via regulating mTORC1. To minimize other variables, we used a randomized complete block design, uniform management (e.g., milking schedules), and Holstein dairy cows of similar genetic background, body weight, and lactation stage (119 ± 26 days in milk). As adequate dietary protein supply may fully activate mTORC1 and hence cover the effects of EAA supplementation, we composed a high-protein diet as a positive control and EAAs were supplemented to each of the two low-protein diets with 12% and 14% CP, respectively. The CP provided by the positive control diet (16% CP) was calculated to meet the protein requirement of a 680 kg BW mid-lactation cow ingesting 24.5 kg/d DM and producing 35 kg/d milk with 3.5% true milk protein content, according to NASEM (2021) [11]. While it is not possible to definitively determine whether the 16% CP diet was truly adequate based on the study design, it was clear that the 12% CP diet was insufficient in protein supply, as evidenced by the lost milk protein production (Table 4).

Both milk protein yield and dietary protein efficiency were improved by supplementing EAAs to the 12% CP diet. Cows receiving this treatment ingested less dietary protein (Table 3, *p* < 0.01), produced similar milk protein (Table 4, *p* = 0.19), and hence had higher dietary nitrogen efficiency (Table 5, *p* < 0.01) compared to those receiving the 16% CP treatment. For cows receiving the 14% CP + EAA treatment, the dietary protein intake and milk protein yield did not show statistical differences from those receiving the 16% CP treatment, but their dietary nitrogen efficiency significantly improved. These positive effects may be attributed to the joint EAA supplementation. Ile, Leu, Met, and Thr were found to have effects on casein synthesis [7] and they further stimulated casein synthesis in mammary tissue slices [4]. mTOR shows a strong sensitivity to Ile and Leu, which in turn effectively passes this signal on to casein synthesis [17]. Adding Ile, Leu, Thr, or Met individually to a 15% CP diet in lactating mice promoted litter weight gain through increased mTOR phosphorylation levels [8]. A subsequent study in Holstein cows demonstrated that co-supplementing RP-Ile, -Leu, -Met, and -Thr restored the 12% CP reduction in milk protein yield through enhanced mTOR signaling [9]. Additionally, the nitrogen balance data (Table 5) further demonstrate the impact of EAA supplementation on nitrogen efficiency. Milk N secretion was significantly decreased in the 12% CP group compared to the 16% CP group. However, adding EAAs to both low-CP diets restored milk N secretion to levels comparable to or exceeding those of the 16% CP group, particularly in the 14% CP + EAA group. Although urinary N excretion was increased with EAA supplementation relative to the 12% CP group, it remained lower than that observed in the 16% CP group. Thus, milk N as a proportion of N intake % was significantly higher in the 12% CP + EAA group than the 16% CP group, indicating enhanced N utilization. The findings underscore the significance of EAA supplementation in promoting nitrogen allocation for milk protein synthesis instead of excretion. These results suggested that dietary supplementation with mammary mTORC1-stimulating EAAs is a promising technique to improve dietary protein efficiency in lactating dairy cows. However, the relationship between mammary translational signaling and milk protein yield did not appear to hold up following longer infusions or treatments, and mechanisms other than phosphorylation of translation factors are responsible for the long-term nutritional effects on milk protein yield [10]. Before these results are recommended for practical use, we need more studies to ascertain the positive effects observed in the present study and studies to evaluate the possible negative effects of the technique, as the estimated nitrogen balances of the 12% CP + EAA and 14% CP + EAA treatments were negative (−62.50 and −23.79 g/d, respectively), which may be harmful to cow health over long time periods. Theoretically, a sustained negative nitrogen balance could lead to muscle protein catabolism or compromised immune function, potentially affecting cow longevity and productivity, although these risks remain speculative without long-term data.

Although milk protein yield was restored in the 12% CP + AA treatment, fiber digestibility remained a noteworthy issue. Table 3 shows that NDF and ADF digestibilities were significantly lower in the 12% CP + AA diet (40.98% and 36.59%, respectively) compared to the 16% CP positive control (49.33% and 44.53%, respectively). The roughage compositions were kept constant in all diets, and the protein levels were adjusted via the concentrate portion (Table 1). The 12% CP diet contained more corn grain and less soybean meal. This change may have altered rumen fermentation kinetics and reduced fiber digestibility, which is often associated with reduced energy utilization and milk protein production. However, milk protein production in the 12% CP + AA treatment was comparable to the 16% CP level (*p* = 0.19; Table 4). This suggests that the NDF and ADF digestibility limitation may have been offset to some extent by the enhanced mammary protein synthesis driven by Met, Ile, Leu and Thr, although further research is needed to confirm this interaction and its long-term effects.

Although the rationale of the present study was based on the hypothesis that supplementation with these EAAs would stimulate mTORC1 and enhance the milk protein response, systemic responses, such as secretion of pancreatic hormones, may play a part in mediating the observed responses to the current study. Variations in extracellular insulin and amino acids can be sensed and integrated by the intracellular mTORC1 pathway but through different mechanisms [18]. It is well documented that elevated circulating insulin promotes milk protein synthesis [19,20,21,22]. The decreased circulating insulin associated with supplementation with the four EAAs to the 12% CP diet might, therefore, exert negative impacts on milk protein synthesis. Contrary to what was anticipated with decreased circulating insulin, cows in this treatment (12% CP + EAA) produced more milk protein than those in the 12% CP treatment. A possible explanation is that the mTORC1-stimulating effects of the supplemented EAAs outweighed the negative effects of decreased circulating insulin on milk protein synthesis. However, the underlying mechanism remains to be elucidated. Circulating insulin has been shown to be affected by the availability of specific amino acids. In lactating rats, circulating insulin was negatively related to circulating Lys but positively related to circulating Thr, Val, Met, and Phe [23]. Arg infusion invoked a dramatic but transient increase in circulating insulin in lactating cows [24]. Among the 17 individual amino acids that have been intravenously infused in sheep, circulating insulin was elevated by Leu, Ala, Gly, or Ser, with Leu having the strongest effect [25]. The insulin-depressing effect of the EAAs in the present study seemed to be affected by dietary CP, as it was not observed in the 14% CP + EAA treatment. Compared to the 14% CP + EAA treatment, the concentration of EAA-LIMTs (i.e., essential amino acids other than the four supplemented ones: Leu, Ile, Met, and Thr) was significantly decreased, perhaps explaining the decline in circulating insulin for the 12% CP + EAA treatment.

Changes in circulating concentrations of other EAAs may also have mitigated the milk protein response to the four EAAs that were supplemented. Lys and His have been shown to influence milk protein synthesis under certain dietary conditions (NASEM, 2021) [11]. Thus, the significant decreases in Lys concentration in the 12% CP and 12% CP + EAA treatments may have partly attenuated the positive effects of the supplemented EAAs. Additionally, the decline in Arg, His, Phe, and Val concentrations for those 2 treatments may have negatively affected the responses. Low Val supply could be limiting for the synthesis of milk protein [26]. Increasing the post-ruminal supply of Arg through jugular perfusion has been shown to positively influence milk protein synthesis [27]. Furthermore, other EAAs beyond Ile, Leu, Met, Lys, and His may be critical in optimizing dairy cattle performance [28]. Recent meta-analysis work supports the positive effects of Arg and His, along with Ile, Leu, Lys, and Met, on milk protein production in lactating cows (Hanigan et al., under review).

## 5. Conclusions

The dietary protein efficiency of lactating cows can be improved by decreasing dietary protein levels, but the milk protein yield generally decreases concomitantly. This study indicated that supplementing low-protein diets with a combination of rumen-protected Leu, Ile, Met, and Thr was a promising strategy to improve the dietary protein efficiency of lactating cows without a concomitant reduction in milk protein yield. For the cows involved in this study, the appropriate dietary CP level was 16%, but reducing it to 12% CP with EAA supplementation maintained milk protein yield, while a 14% CP + EAA diet even achieved numerically higher yields. Although these results are encouraging, their practical application requires caution, as longer-term studies are essential to confirm the efficacy and assess the potential health impacts, such as those from negative nitrogen balances observed in this study. Pending further validation, the 14% CP + EAA diet may represent an optimal balance of efficiency and performance for practitioners, offering a practical foundation for future research and application.

## Figures and Tables

**Table 1:** Ingredients and composition of the experimental diets.

Item	Dietary CP (%), AA (g/d)			
	16%CP	12%CP	12%CP + AA	14%CP + AA
Ingredient, % of DM				
Corn meal	2.14	2.14	2.14	2.16
Corn grain, ground	18.24	27.09	27.09	21.12
Soybean meal	13.66	4.87	4.87	10.59
Corn silage	31.30	31.29	31.29	31.35
Wheat bran	3.87	3.88	3.88	3.87
Whole cottonseed	3.25	3.25	3.25	3.26
Oaten hay	3.74	3.87	3.87	3.91
Alfalfa hay	15.96	15.75	15.75	15.89
Brewers grains	3.67	3.67	3.67	3.67
Hydrogenated vegetable fat product	0.61	0.61	0.61	0.61
Minerals and vitamins premix ^1^	3.57	3.57	3.57	3.57
Rumen-protected Met (RP-Met), ^2^ g/d	-	-	16.71	9.28
Rumen-protected Leu (RP-Leu), ^3^ g/d	-	-	66.78	33.39
Rumen-protected Ile (RP-Ile), ^4^ g/d	-	-	55.54	24.90
Rumen-protected Thr (RP-Thr), ^5^ g/d	-	-	103.31	47.35
Nutrient level, %, DM basis				
CP ^8^	16.0	12.4	12.4	8
RDP ^6^	10.8	8.1	8.1	9.9
RUP ^6^	5.2	4.4	4.4	4.9
Microbial Protein ^6^	1.93	1.56	1.56	1.76
MP ^6^	9.38	7.79	7.79	8.87
NE_L_, Mcal/kg ^6^	1.72	1.73	1.73	1.73
Ether extract	3.34	3.52	3.52	3.41
NDF	33.1	32.9	32.9	33.1
ADF	20.6	20.2	20.2	20.5
NFC ^7^	42.1	46.2	46.2	43.4
Ash	5.5	5	5	5.4
Starch	27.2	35.1	35.1	29.8
dMet from RP-Met, g/d	-	-	9	5
dLeu from RP-Leu, g/d	-	-	36	18
dIle from RP-Ile, g/d	-	-	29	13
dThr from RP-Thr, g/d	-	-	24	11

^1^ The premix contained (%, as-is basis): VA, 260 KIU/kg; VD3, 50 KIU/kg; VE, 300 mg/kg; P, 84 mg/kg; Mn, 276 mg/kg; Zn, 1032 mg/kg; Salt, 80 mg/kg; Ca, 200 mg/kg; Cu, 380 mg/kg; Fe, 180 mg/kg. ^2^ The RP-Met product contained 65.56% methionine and had 82% bioavailability (90.9% rumen escape and 90.4% intestinal digestibility). ^3^ The RP-Thr product contained 31.78% threonine and had 73% bioavailability (86.2% rumen escape and 84.8% intestinal digestibility). ^4^ The RP-Leu product contained 70.04% leucine and had 77% bioavailability (89.4% rumen escape and 86.1% intestinal digestibility). ^5^ The RP-Ile product contained 65.4% isoleucine and had 80% bioavailability (88.9% rumen escape and 89.8% intestinal digestibility). ^6^ Calculated data from NASEM (2021) [11]. ^7^ NFC = 100 − (% NDF + % CP + % ether extract + % ash). ^8^ Contributions of supplemented RP-AAs to dietary CP content were not included. Including the contribution of supplemented RP-AAs, the CP contents of the 12%CP + AA and 14%CP + AA diets were 12.4% and 14.7% DM, respectively.

**Table 2:** Duodenal flows of digestible EAAs as predicted by NASEM (2021).

EAA	Diet ^1^							
	16%CP		12%CP		12%CP + AA ^2^		14%CP + AA	
	AA Flow,	MP,	AA Flow,	MP,	AA Flow,	MP,	AA Flow,	MP,
	g/d	%	g/d	%	g/d	%	g/d	%
Arg	131	5.66	99	5.33	99	5.07	117	5.47
His	56	2.42	45	2.42	45	2.30	50	2.34
Ile	135	5.83	106	5.71	135	6.91	135	6.31
Leu	213	9.20	177	9.54	213	10.90	213	9.95
Lys	171	7.39	131	7.06	131	6.70	153	7.15
Met	51	2.20	42	2.26	51	2.61	51	2.38
Phe	133	5.75	106	5.71	106	5.42	120	5.61
Thr	121	5.23	97	5.23	121	6.19	121	5.65
Trp	30	1.30	23	1.24	23	1.18	27	1.26

Abbreviations: EAA = essential amino acid; MP = metabolizable protein; CP = crude protein. ^1^ MP contents of the 16%CP, 12%CP, 12%CP + AA, and 14%CP + AA diets were 2314, 1856, 1954, and 2140 g/d, respectively. ^2^ Ratios of specific RP-EAAs to MP in the supplemented diet were calculated as follows: (the content of the individual EAA in the 16%CP diet)/[content of MP in 12%CP diet + (the content of the individual EAA in the 16%CP diet—the content of that EAA in the 12%CP diet)].

**Table 3:** Effects of dietary CP level and supplementation with a mixture of ruminally protected Ile, Leu, Met, and Thr on nutrient intake and apparent digestibility in lactating Holstein cows.

	Dietary CP (%), AA				SEM ^1^	Contrast ^2^				
	16	12	12 + AA	14 + AA		16 vs. 12	12 + AA vs. 12	12 + AA vs. 16	14 + AA vs. 16	14 + AA vs. 12 + AA
Nutrient intake, kg/d										
DM	24.66	23.81	24.23	24.67	0.254	0.46	0.44	0.47	1.00	0.88
OM	22.78	21.98	22.36	22.78	0.233	0.45	0.43	0.45	1.00	0.87
CP	3.87	3.07	3.15	3.70	0.061	<0.01	<0.01	<0.01	0.19	<0.01
NDF	7.86	8.17	8.35	8.05	0.083	0.38	0.92	0.02	0.75	0.08
ADF	4.78	4.98	5.09	4.88	0.051	0.34	0.83	0.01	0.82	0.06
Apparent digestibility ^3^, %										
DM	67.21	60.49	61.04	64.72	0.753	<0.01	0.12	<0.01	0.54	0.01
OM	69.40	62.85	62.44	67.15	0.731	<0.01	0.08	<0.01	0.58	<0.01
CP	67.95	57.00	58.00	62.06	0.982	0.05	<0.01	<0.01	0.13	<0.01
NDF	49.33	40.74	40.98	46.67	1.174	0.03	0.22	<0.01	0.82	0.05
ADF	44.53	37.09	36.59	43.95	1.295	0.15	0.19	0.03	0.99	0.12

^1^ Largest SEM published in table. ^2^ *p*-values for the main effect of treatment. ^3^ Urinary excretion was estimated using creatinine as a volume marker, while fecal excretion was assessed through acid-insoluble ash (AIA) as an internal marker.

**Table 4:** Effects of dietary CP level and supplementation with a mixture of ruminally protected Ile, Leu, Met, and Thr on milk yield and composition in lactating Holstein cows.

Item	Dietary CP (%), AA				SEM ^1^	Contrast ^2^				
	16	12	12 + AA	14 + AA		16 vs. 12	12 + AA vs. 12	16 vs. 12 + AA	16 vs. 14 + AA	14 + AA vs. 12 + AA
Milk yield, kg/d	34.88	31.39	34.72	34.95	0.572	0.03	0.04	0.93	0.97	0.13
DMI, kg/d	24.77	23.81	24.23	24.66	0.251	0.47	0.45	0.47	1.00	0.88
Feed efficiency, ^3^ kg/kg	1.40	1.32	1.44	1.42	0.023	0.17	0.04	0.63	0.82	0.67
Milk protein, %	3.80	3.54	3.60	3.81	0.038	<0.01	0.58	0.04	0.91	<0.01
kg/d	1.32	1.13	1.24	1.33	0.024	<0.01	<0.01	0.19	1.00	0.55
Milk fat, %	4.42	4.41	4.26	4.67	0.094	1.00	0.74	0.57	0.76	0.94
kg/d	1.54	1.40	1.47	1.63	0.041	0.63	0.18	0.56	0.84	0.93
Milk lactose, %	5.05	5.06	5.04	4.99	0.018	1.00	0.64	0.86	0.73	1.00
kg/d	1.76	1.59	1.77	1.74	0.032	0.05	0.05	0.96	0.86	0.83
Milk SNF, %	9.07	8.86	8.81	9.04	0.283	0.29	0.37	<0.01	0.97	0.05
kg/d	3.16	2.79	2.64	3.16	0.152	0.06	0.86	0.60	0.99	0.08
Milk TS, %	13.49	13.27	13.07	13.72	0.101	0.44	0.47	0.14	0.41	0.02
kg/d	4.70	4.19	4.56	4.79	0.153	0.03	0.05	0.53	0.69	0.05
ECM, ^4^ kg/d	38.53	34.47	37.08	39.63	0.754	0.05	0.06	0.49	0.58	0.22
ECM feed efficiency, ^5^ kg/kg	1.55	1.45	1.53	1.60	0.028	0.12	0.13	0.65	0.54	0.29
Milk NE_L_, ^6^ Mcal/d	28.70	25.73	27.66	29.58	0.563	0.05	0.06	0.49	0.58	0.22
MUN, mg/dL	13.67	9.27	10.31	11.77	0.292	<0.01	<0.01	<0.01	<0.01	<0.01

^1^ Largest SEM published in table. ^2^ *p*-values for the main effect of treatment. ^3^ Feed efficiency = Milk yield/DMI. ^4^ Energy-corrected milk (kg/d) = kg of milk × [(38.3 × % fat × 10 + 24.2 × % true protein × 10 + 16.54 × % lactose × 10 + 20.7) ÷ 3140]. ^5^ ECM feed efficiency = ECM/DMI. ^6^ Milk NE_L_ (Mcal/d) = kg of milk × (0.0929 × % fat + 0.0563 × % true protein + 0.0395 × % lactose) [11].

**Table 5:** Effects of dietary CP level and supplementation with ruminally protected Ile, Leu, Met, and Thr on nitrogen balance in lactating Holstein cows.

	Dietary CP (%), AA				SEM ^1^	Contrast ^2^				
	16	12	12 + AA	14 + AA		16 vs. 12	12 + AA vs. 12	12 + AA vs. 16	14 + AA vs. 16	14 + AA vs. 12 + AA
N intake, g/d	610	490	510	590	9	<0.01	0.13	<0.01	0.15	<0.01
N secretion and excretion, g/d										
Milk N	202.8	177.7	199.4	212.8	3.62	<0.01	0.01	0.71	0.29	0.10
Urinary N	212.9	154.9	166.1	191.2	4.43	<0.01	0.20	<0.01	0.01	<0.01
Fecal N	193.3	211.4	208.6	206.0	4.31	0.14	0.82	0.21	0.30	0.83
As proportion of N intake, %										
Milk N	33.3	36.5	39.0	36.3	0.57	<0.01	0.40	<0.01	0.04	0.13
Urinary N	34.9	31.8	32.5	32.6	0.59	0.09	0.76	0.17	0.21	0.91
Fecal N	31.7	43.4	40.8	35.1	1.03	<0.01	0.19	<0.01	0.41	0.02
Estimated N balance, g/d	0.8	−57.2	−62.5	−23.8	6.62	<0.01	0.97	<0.01	0.14	0.03

^1^ Largest SEM of the individual treatment estimates. ^2^ *p*-values for the main effect of treatment.

**Table 6:** Effects of dietary CP level and supplementation with a mixture of ruminally protected Ile, Leu, Met, and Thr on plasma free amino acids (μmol/L) in lactating Holstein cows.

	Dietary CP (%), AA				SEM ^1^	Contrast ^2^				
	16	12	12 + AA	14 + AA		16 vs. 12	12 + AA vs. 12	12 + AA vs. 16	14 + AA vs. 16	14 + AA vs. 12 + AA
Arg	81.9	74.00	69.0	81.7	1.88	0.11	0.52	0.03	0.64	<0.01
His	49.3	41.4	42.2	52.0	1.45	0.05	0.82	0.09	0.53	0.02
Ile	118	96.5	105	111	2.2	<0.01	0.06	0.04	0.24	0.37
Leu	158	132	139	150	3.3	<0.01	0.16	0.07	0.40	0.33
Lys	91.2	77.5	77.1	86.1	1.93	0.03	0.98	0.02	0.43	0.14
Met	24.4	22.2	24.2	24.7	0.36	0.01	0.01	0.96	0.73	0.69
Phe	42.0	38.4	37.6	40.2	0.63	0.04	0.81	0.02	0.41	0.13
Thr	79.6	78.6	79.1	79.4	1.42	0.69	0.59	0.90	0.99	0.89
Val	253	204	212	236	5.3	<0.01	0.42	0.04	0.33	0.09
EAA	898	765	784	861	15.2	<0.01	0.36	0.02	0.47	0.08
EAA-LIMT	517	435	438	496	9.6	<0.01	0.73	<0.01	0.55	0.03
Ala	193	204	204	197	3.2	0.27	0.40	0.05	0.50	0.20
Asp	20.5	19.2	19.0	18.2	0.46	0.24	0.67	0.43	0.18	0.55
Cys	1.60	1.66	1.56	1.52	0.031	0.45	0.26	0.68	0.36	0.62
Glu	85.2	88.2	86.3	82.8	1.58	0.38	0.62	0.68	0.92	0.60
Gly	221	279	262	238	6.3	<0.01	0.51	<0.01	0.10	0.16
Ser	69.3	76.0	68.0	69.0	1.53	0.12	0.21	0.75	0.54	0.76
Tau	34.2	33.6	30.6	31.2	0.78	0.56	0.57	0.25	0.17	0.81
Tyr	54.6	48.4	48.5	50.5	1.13	0.09	0.63	0.03	0.29	0.26
NEAAs	680	750	720	688	9.7	<0.01	0.61	0.01	0.27	0.16

Abbreviations: EAA = essential amino acid; LIMT = EAA other than the four supplemental EAAs (Met, Thr, Ile, Leu); NEAAs = non-essential amino acids. ^1^ Largest SEM published in the table. ^2^ *p*-values for the main effect of treatment.

**Table 7:** Effects of dietary CP level and supplementation with a mixture of ruminally protected Ile, Leu, Met, and Thr on plasma metabolites and hormones in lactating Holstein cows.

Item	Dietary CP (%), AA				SEM ^1^	Contrast ^2^				
	16	12	12 + AA	14 + AA		16 vs. 12	12 + AA vs. 12	12 + AA vs. 16	14 + AA vs. 16	14 + AA vs. 12 + AA
Total protein, g/L	75.66	74.67	78.14	75.81	1.478	0.82	0.42	0.57	0.97	0.58
Urea, mmol/L	4.32	2.34	2.84	4.28	0.143	<0.01	<0.01	<0.01	0.85	<0.01
Glucose, mmol/L	3.50	3.65	3.57	3.68	0.052	0.31	0.60	0.59	0.19	0.42
Nitric oxide, μmol/L	106.25	87.82	73.06	105.83	8.237	0.45	0.53	0.16	0.99	0.16
Insulin, μIU/mL	25.75	23.14	16.85	23.74	1.114	0.39	0.04	<0.01	0.50	0.02
Glucagon, pg/mL	102.12	101.87	120.34	109.27	3.513	0.78	0.04	0.07	0.45	0.25
Growth hormone, ng/mL	1.19	1.06	1.24	1.38	0.052	0.34	0.20	0.71	0.15	0.28
IGF-I, mg/mL	96.51	88.67	104.45	111.09	6.214	0.66	0.38	0.65	0.41	0.71
Prolactin, ng/mL	10.90	10.15	12.04	11.91	0.245	0.11	<0.01	0.07	0.21	0.84

^1^ Largest SEM published in the table. ^2^ *p*-values for the main effect of treatment.

## Data Availability

The original contributions presented in this study are included in the article. Further inquiries can be directed to the corresponding author.

## Abbreviations

The following abbreviations are used in this manuscript:

EAA	Essential amino acid
CP	Crude protein
DM	Dry matter
DMI	Dry matter intake
NDF	Neutral detergent fiber
ADF	Acid detergent fiber
AIA	Acid insoluble ash
IGF-I	Insulin-like growth factor
GH	Growth hormone
BW	Body weight
MUN	Milk urea nitrogen
ECM	Energy-corrected milk
mTOR	Mammalian target of rapamycin
